# The effect of supplemental high Fidelity simulation training in medical students

**DOI:** 10.1186/s12909-020-02322-y

**Published:** 2020-11-10

**Authors:** Lori Meyers, Bryan Mahoney, Troy Schaffernocker, David Way, Scott Winfield, Alberto Uribe, Ana Mavarez-Martinez, Marilly Palettas, Jonathan Lipps

**Affiliations:** 1 Department of Anesthesiology, The Ohio State University Wexner Medical Center, Columbus, OH USA; 2 Department of Anesthesiology, Perioperative and Pain Medicine, Mount Sinai West and Mount Sinai Morningside Hospitals, Icahn School of Medicine at Mount Sinai, New York, NY USA; 3 Department of Internal Medicine, The Ohio State University Wexner Medical Center, Columbus, OH USA; 4 Department of Emergency Medicine, The Ohio State University Wexner Medical Center, Columbus, OH USA; 5 The Ohio State University, College of Medicine: Clinical Skills Education and Assessment Center, EDU-Medicine Education, Columbus, OH USA; 6 Department of Biomedical Informatics, Center for Biostatistics, The Ohio State University, Columbus, OH USA

**Keywords:** Simulation-based education, Lecture-based education, High-fidelity simulation, Pulmonary physiology, First-year medical students

## Abstract

**Background:**

Simulation-based education (SBE) with high-fidelity simulation (HFS) offers medical students early exposure to the clinical environment, allowing development of clinical scenarios and management. We hypothesized that supplementation of standard pulmonary physiology curriculum with HFS would improve the performance of first-year medical students on written tests of pulmonary physiology.

**Methods:**

This observational pilot study included SBE with three HFS scenarios of patient care that highlighted basic pulmonary physiology. First-year medical students’ test scores of their cardio-pulmonary curriculum were compared between students who participated in SBE versus only lecture-based education (LBE). A survey was administered to the SBE group to assess their perception of the HFS.

**Results:**

From a class of 188 first-year medical students, 89 (47%) participated in the SBE and the remaining 99 were considered as the LBE group. On their cardio-pulmonary curriculum test, the SBE group had a median score of 106 [IQR: 97,110] and LBE group of 99 [IQR: 89,105] (*p* < 0.001). For the pulmonary physiology subsection, scores were also significantly different between groups (*p* < 0.001).

**Conclusions:**

Implementation of supplemental SBE could be an adequate technique to improve learning enhancement and overall satisfaction in preclinical medical students.

## Background

The preclinical core of medical education has traditionally been based on lectures and textbooks [1]. Recently, significant developments in technology (computers, regular patients, and high-fidelity patient simulators) and research during the last two decades have contributed to the implementation of simulation-based education (SBE) in the medical field [1]. One strategy for improving learning efficiency was the concept of vertical integration—the blending of basic science principles with practical clinical scenarios in order to facilitate the transition from lectures learning to clinical training [2, 3]. However, for practical reasons, learning within a clinical environment is not always possible. The advancements in simulation technology can create a pseudo-clinical and low-stakes environment in which medical students can learn basic scientific concepts and implement their knowledge in pre-clinical and clinical settings [1, 4–10].

A high-fidelity simulation (HFS) manikin makes this kind of learning possible and improves the likelihood that students will retain and make use of their basic scientific knowledge in a pre-clinical setting. SBE is a novel technique that substitutes and magnifies real medical events using guided experiences. These experiences simulate a real medical environment in an interactive style to aid students’ understanding of relevant topics [11, 12].

SBE offers medical students early exposure to the clinical environment which can allow them to develop crisis and management skills, while also learning in an intellectually safe and low-risk environment [13]. HFS for medical students has been added via small group learning in a few medical schools’ curricula [14]. Several simulation modalities have been implemented to teach medical residents in several fields, especially those in high-risk fields (anesthesiology, trauma, emergency, intensive care, and cardiology). The current unfavorable perception of SBE in undergraduate medical education could be attributed to the lack of adequate patient risk environment with real-time physiologic response [1, 13]. Despite this view, SBE improves student engagement and promotes learning amidst emotional stress, also allowing the student to receive immediate feedback and perform unlimited repetitions to ensure enhanced retention of the taught topic [1, 13]. Finally, SBE can alleviate the necessity for enhancing didactic knowledge retention solely through clinical practice. It can compensate for the decreased number of hospital working hours for medical students and help address the ethical concerns surrounding patient safety by allowing students to hone their skills in a simulated environment prior to engaging with patients in the hospital [11, 13].

All the aforementioned features of SBE contribute to making this learning modality a unique method that researchers have been exploring to enhance the learning of undergraduate medical students [1]. A significant proportion of medical knowledge is not retained for extended periods of time among medical students due to its lack of daily use [11]. Therefore, medical education is paired with clinical experience as a technique to improve knowledge retention. However, this clinical experience has been affected by a recent reduction of students’ clinical hours because of uncommon relevant cases and ethical considerations related to safety concerns during clinical practice [11]. The implementation of case-based discussion in concert with HFS may complement clinical practice and knowledge retention. A HFS manikin is a computerized full-body manikin programmed with accurate physiological responses that mimics real clinical situations [15]. There is limited literature comparing SBE and standard lecture-based education (LBE) in pre-clinical medical students, yet the results demonstrate small or moderately significant differences [1, 11].

The purpose of this observational pilot study was to compare enhanced learning acquisition of pre-clinical topics among first-year medical students that participated in a supplementary SBE versus only LBE modalities as part of an organized organ-system curriculum. We hypothesized that the supplementation of standard pulmonary physiology curriculum with HFS would improve the first-year medical student’s performance on written tests of pulmonary physiology.

## Methods

### Trial design

**Fig. 1:**
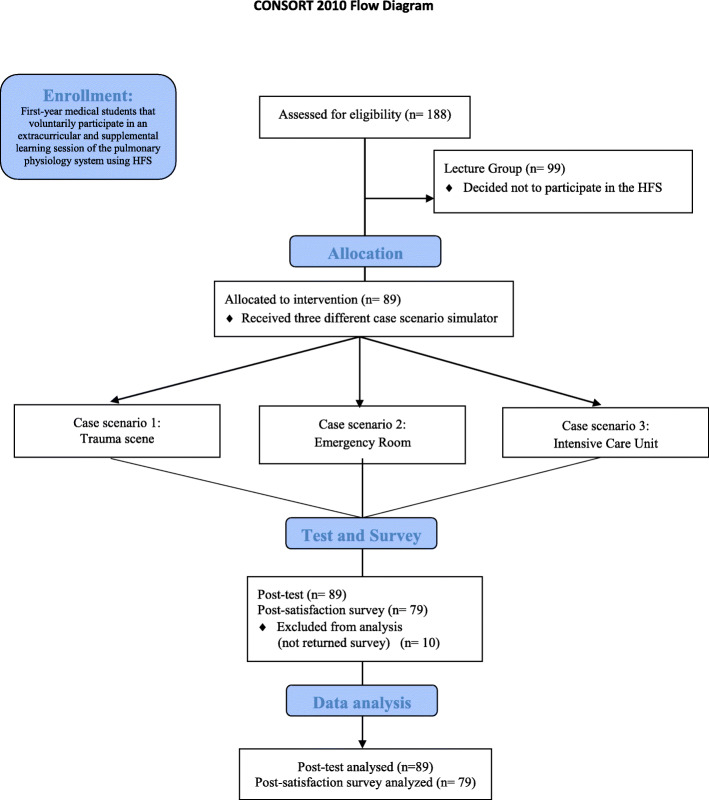
CONSORT Enrollment Flow Chart

A single center non-randomized two-group observational pilot trial design was conducted according to the CONSORT statement – Simulation-based research extension **(**Fig. 1**)** [16]. A supplemental HFS is usually offered to all first-year medical students as part of their basic science curriculum. However, during 2014, this observational pilot study was designed to be an optional (volunteer) activity for them. The study (2015E0170) was reviewed by our Institutional Review Board (Office of Responsible Research Practices-The Ohio State University) and approved as an exempt study (category #1) with informed consent waiver due to the observational nature of the study and the involvement of normal educational practices. Therefore, participants were not consented because they were participating in their regular and special education instructional strategies as part of the standard curricula. All students who participated in the optional HFS session of pulmonary physiology were allocated to the simulation group. Four sessions per day were offered during one week in April 2014. Each session was limited to eight medical students. The remaining students who did not volunteer to participate in the HFS sessions were allocated to the LBE group (control).

### Participants

First-year medical students from The Ohio State University, College of Medicine who were participating in a pulmonary curriculum block were offered to participate in an extracurricular and supplemental learning session of the pulmonary physiology system using HFS. Recruitment was done on a voluntary basis. Students were told about the simulation sessions through announcements in class and by email and were provided with a schedule link in Google Docs or in person at the Office of Medical Education where they were able to sign up for the sessions. MCAT scores were collected and compared between groups to assess academic baseline characteristics.

### Interventions

The faculty instructors in charge of this observational pilot study created a novel 75 min session with multiple case scenario simulations that included three separate 25 min stations each simulating different phases of patient care which highlighted basic pulmonary concepts and physiology. Students started with a basic pulmonary physical examination and progressed to interpretation of external and invasive hemodynamic monitoring to integrate basic science concepts with clinical applications.

The first station took place in the simulation center hallway; this was a trauma scene involving an unresponsive patient lying on the floor. The patient was portrayed by a Laerdal SimMan® 3G manikin. At this station, students were taught how to evaluate the pulmonary function of the patient without medical equipment.

The second station was a simulated emergency room trauma bay; the trauma patient was portrayed by a CAE Healthcare HPS® manikin. Medical students were taught how to properly place and read the monitors to assess the pulmonary system. The manikin was preprogrammed to simulate clinical scenarios of tension pneumothorax, a right-mainstem intubation and atelectasis. With the aid of the electronic SMART board, topics such as oxygenation, the alveolar gas equation, ventilation/perfusion (V/Q) mismatch (shunt), and lung volumes were explained. Following the clinical scenario, medical students were given the opportunity to treat the patient as they would in a real clinical environment.

The third station was a simulated intensive care unit (ICU) where the patient was portrayed using a Laerdal SimMan® 3G manikin. In the ICU, the focus was mainly on the principles of ventilation, dead space, and the West's zones of the lung. Three faculty instructors mentored the medical students through the three scenarios, with one being present at each station. At each station, the faculty instructor described the setting and guided the student participants through the patient case. Rather than one debriefing session at the end of the clinical scenario, briefer “time outs” were conducted throughout the case scenario in order to reinforce the important learning objectives and to offer time for questions and clarifications.

An instructor facilitator that represented a health care provider was also present piloting the three scenarios. The facilitator’s primary role was to assure the chronological changes in the patient’s status according to the evolution of the case and alert medical students regarding undetected changes. In addition, a simulator operator was also sitting behind a two-way glass and was responsible for performing the mechanical changes on the simulator according to the scripts and cues from the faculty facilitator. During the time-outs, a debriefing for meaningful learning (DML) process was developed, students and instructors systematically processed the test results or data on the patient’s condition from the monitors in order to generate critical thinking from new concepts and debrief different aspects of reflection from the simulation experience. Questions from the instructor helped the students to interpret the data and make patient management decisions based on their interpretations.

### Outcomes

The pulmonary simulation sessions took place approximately five weeks prior to the final examination over the cardio-pulmonary curriculum block. At the end of the block, students took their curricular test covering anatomy, physiology, and pathophysiology of the cardio-pulmonary system from regular lectures. An overall pulmonary score from 51 items was compared between SBE and LBE groups. After the block concluded, faculty instructors that conducted the HFS reviewed the examination and found that six questions had unambiguous teachable material included in the HFS pulmonary physiology scenarios. These six topics were stratified and scored separately from the overall test score and compared between groups. Raw scores on these items were converted to percentage correct scores.

Additionally, at the conclusion of each session, medical students from the simulation group completed an anonymous eight-point Likert scale satisfaction survey in order to assess their perception of the utility of the HFS session.

### Statistical methods

Test scores and survey responses were summarized using descriptive statistics. Test scores, which were not normally distributed, were expressed using medians and interquartile range (IQR) and survey responses were expressed using frequencies and proportions. To evaluate student performance at the end of unit examination, comparisons in test scores between SBE group and LBE group were assessed using descriptive statistics as well as a Kruskal-Wallis test, where appropriate. Statistical analysis was performed using SAS/STAT statistical software (version 9.4 of SAS for Windows, SAS Institute Inc., Cary, NC).

## Results

### Recruitment

A total of 89 (47%) from a class of 188 first-year medical students participated in the HFS of pulmonary physiology and were allocated to the SBE group. The remaining 99 medical students were considered as the comparison group and allocated to the LBE group.

### Baseline data

Students’ baseline median MCAT score was 33 [IQR: 31, 36] for the SBE group and 34 [IQR: 32, 36] for the LBE group, with no significant difference between the two groups (*p* = 0.1372).

### Outcomes and estimation

The test at the end of students’ cardio-pulmonary curriculum block evaluated their global cardio-pulmonary knowledge. The SBE group had a median score of 106 [IQR: 97, 110] and the LBE group scored 99 [IQR: 89, 105] (*p* < 0.001). Further stratification of the test by general pulmonary physiology questions showed a significant difference between both groups (44 [IQR: 42, 47] vs 42 [IQR: 38, 44]; p < 0.001). Finally, pulmonary physiology questions as instructed in the HFS session were subdivided and compared, showing significant difference as well (5 [IQR: 4, 6] vs 5 [IQR: 4, 5] *p* = 0.014) **(**Table 1**).**

**Table 1:** Test scores after high fidelity simulation participation

Test scores	SBE Group Median [IQR] ***N*** = 89	LBE Group Median [IQR] ***N*** = 99	***P***-value
**Global cardio-pulmonary test**			
Score	106 [97,110]	99 [89,105]	< 0.001
% score	87.6 [80,90.9]	81 [74,86.8]	< 0.001
**Pulmonary Physiology section**			
Total score	44 [42,47]	42 [38,44]	< 0.001
% score	86.3 [82,92.2]	82.4 [75,86.3]	< 0.001
**Pulmonary physiology from HFS**			
score	5 [4,6]	5 [4,5]	0.014
% score	83.3 [67,100]	83.3 [67,83.3]	0.014

Results of the student rating to their participation in the HFS are presented in Table 2**.** Of the 89 students who participated, 79 (89%) completed the survey.

**Table 2:** Student rating to simulation activity, *n* = 79

	Strongly agree	Agree	Disagree/ Agree equally	Disagree
The session contributed significantly to my understanding of pulmonary physiology	50 (63%)	27 (34%)	1 (1%)	1 (1%)
The session increased my knowledge of the pulmonary system	53 (67%)	25 (32%)	0 (0%)	1 (1%)
I can better explain pulmonary physiology concepts	46 (58%)	27 (34%)	5 (6%)	1 (1%)
Pace was appropriate	53 (67%)	23 (29%)	1 (1%)	2 (3%)
Lab was well organized	61 (77%)	17 (22%)	1 (1%)	0 (0%)
Instructors showed respect for student time	64 (81%)	14 (18%)	1 (1%)	0 (0%)
Instructors effectively addressed student questions	65 (82%)	14 (18%)	0 (0%)	0 (0%)
Instructors taught in a way that students could understand	64 (81%)	14 (18%)	1 (1%)	0 (0%)

## Discussion

### Limitations

There are a few limitations that were identified in this study. Firstly, SBE- is usually based on fidelity of the simulator in order to produce similar results of a real situation; therefore, it might be possible that the situation taught to the medical students couldn’t be reproducible in some clinical scenarios. Secondly, the lack of randomization of the groups and the volunteerism design of the study could have an effect on the results. Consequently, a selection bias could be present during student recruitment because students with higher academic performances may be more prompt to participate in any supplemental educational activity, including SBE, despite observing the lack of statistical difference (*p* = 0.1372) of the MCAT scores from both groups. Lastly, an exploratory pretest-posttest including specific knowledge from the simulation experience was not conducted during the administration of the HFS, leading to the inability of assessing short term and long term knowledge specific for their simulation experience.

### Generalizability

Several studies have demonstrated the equivalence or superiority of HFS as an educational technique to learning enhancement of a taught topic [1, 13]. A summary table of the debriefing evaluation HFS from this study is presented in Table 3. The results from our study are compatible with these results. Learning human physiology concepts in medical school has traditionally revolved around lectures and textbook memorization. While the traditional approach can be effective for many learners, HFS offers an alternative illustration of these concepts that appeal to visual, audio, and kinesthetic learners through multisensory engagement. Teaching physiology through HFS also achieves the goal of vertical curriculum integration, a blending of basic science principles with the clinical setting. While medical students generally embrace simulation as a learning method, the positive experience must be balanced with cost-effectiveness, time efficiency, and assessment results.

**Table 3:** Debriefing Evaluation High-Fidelity Simulation (HFS) in first-year medical students

Elements	Descriptions
Study Purpose	To compare enhanced learning acquisition of pre-clinical topics among first-year medical students that participated in a supplementary simulation-based education (SBE) versus only lecture-based education (LBE) modalities as part of an organized organ-system curriculum.
Study Design	A single center non-randomized two-group trial
Sample Population	First-year medical students (*n* = 188) from The Ohio State University, College of Medicine. Intervention group (*n* = 89) and control group (*n* = 99)
Outcome Variables with Measurement	Enhanced learning acquisition of pre-clinical topics among first-year medical students related to the pulmonary physiology system and student’s overall satisfaction of the debriefing experience
Debriefing Evaluation	The test at the end of students’ cardio-pulmonary curriculum block evaluated their global cardio-pulmonary knowledge. The SBE group had a median score of 106 [IQR: 97, 110] and the LBE group scored 99 [IQR: 89, 105] (*p* < 0.001). Results of the student rating to their participation in the HFS are presented in Table 2. Of the 89 students who participated, 79 (89%) completed the survey.

Simulation is usually reserved for clinical years of medical school; incorporating HFS to preclinical first-year medical students demonstrated that simulation is a feasible tool to integrate basic knowledge with clinical scenarios. Harris et al. evaluated first-year medical students’ knowledge and perception of cardiovascular physiology and congestive heart failure (CHF) treatment strategies. Comparing pretest and posttest scores of a six-question test after simulation demonstrated a 22% increase in correct answers (8). Similarly, Heitz et al. evaluated basic neuroscience concepts on 112 first-year medical students before and immediately after HFS through a four-question multiple choice test. After simulation, 83% of the participants scored the four questions correctly compared to 55% before simulation (6). In our study, instead of using simulation-related test scores, we used the scheduled block curriculum test to evaluate students’ knowledge acquired during simulation within a larger range of topics.

Additionally, instead of comparing same student pretest and posttest scores, our study compared students that participated in the SBE HFS scenarios with students only attending LBE as the control group to assess knowledge improvement after implementation of simulation scenarios. Likewise, Alluri et al. evaluated 20 second-year medical students attending simulation sessions and lecture sessions and compared pretest and posttest for each group. Both groups demonstrated significant improvement after the sessions (*p* = 0.023 for SBE, *p* = 0.001 for LBE), nevertheless, comparison between SBE and LBE groups was not performed, making our study unique (1).

Clear learning objectives must be defined when developing a simulation clinical scenario, focusing on diagnosis, management, and treatment of the targeted clinical situation. Morgan et al. evaluated 299 medical students after HFS that involved clinical scenarios resulting in unstable cardiac arrhythmia; learning objectives were recognition of anaphylaxis under anesthesia, management of anaphylaxis, and treatment of pulseless tachycardia (9). Our study focused HFS on physiologic concepts of pulmonary function; learning objectives were evaluation of pulmonary function without medical equipment, treatment of tension pneumothorax, and management of pulmonary function in the ICU.

Feedback from the simulation participants is essential to enhanced future scenarios and learning tools. Using a five-point Likert scale post-simulation, Heitz et al. showed that 97% of the participants agreed that HFS improved their learning of the neuroscience concepts (6). In the study performed by Harris et al., participants agreed that simulation helped them to learn cardiovascular curves (73%) and CHF treatment strategies (84%) (8). In our study, students who experienced the pulmonary physiology simulation not only confirmed they enjoyed learning through this method of teaching, but also outperformed their peers who did not participate in the sessions on the unit examination.

The investment in faculty time and resources is significant. However, we believe that the positive outcomes justify the expense, particularly since we anticipate that learning human physiology in the clinical context will save time and energy later on during the student’s clinical training. This curricular innovation has also allowed us to achieve a greater degree of vertical curriculum integration. While simulation has previously shown to be a useful tool for teaching basic physiology principles, our study further strengthens the case that high fidelity simulation, carefully scripted to provide a rich clinical context, enhances medical students' understanding of basic pulmonary physiology concepts through multisensory engagement [1, 7–10].

High-fidelity human physiology simulation equipment and qualified technical support staff requires significant institutional monetary investment. We also found faculty support and preparation to be critical to the success of this simulation session. Well trained instructors are needed to maintain the flow of the simulation session, illustrate key teaching points, be prepared to adapt to the varied reactions of the learners, and answer questions.

One option that could be explored is whether similar learning gains would be observed if students watched a recording of this session in lieu of live participation. This could be a study design option for future research that could possibly save faculty and technical resources.

## Conclusions

In conclusion, our results suggest that the implementation of supplemental SBE could be an adequate technique to improve learning enhancement and overall satisfaction in preclinical medical students. Future studies should implement this study as part of a randomized design study in order to explore more the efficacy of the use of SBE in preclinical medical students.

### Practice points

- Pulmonary physiology lectures in the preclinical setting can be combined with simulation scenarios with high-fidelity simulation for increased learning enhancement.
- First-year medical students reported that simulation-based education increased their knowledge of the pulmonary system.
- Implementation of simulation scenarios in early years of medical school offers students timely exposure to the clinical environment.

## Data Availability

The database generated and/or analyzed by during the current study are available from the corresponding author on reasonable request.

## Abbreviations

CHF: Congestive heart failure
HF: High-fidelity simulation
ICU: Intensive care unit
IQR: Interquartile range
SBE: Simulation-based education
